# Full-length transcriptome sequencing analysis and development of EST-SSR markers for the endangered species *Populus wulianensis*

**DOI:** 10.1038/s41598-020-73289-5

**Published:** 2020-10-01

**Authors:** Qichao Wu, Fengqi Zang, Xiaoman Xie, Yan Ma, Yongqi Zheng, Dekui Zang

**Affiliations:** 1 College of Forestry, Key Laboratory of State Forestry Administration for Silviculture of the Lower Yellow River, Shandong Agricultural University, Tai’an, 271018 People’s Republic of China; 2 Institute of Forestry, Chinese Academy of Forestry, Beijing, 100091 People’s Republic of China; 3 Center for Forest Genetic Resources of Shandong Province, Jinan, 250014 People’s Republic of China

**Keywords:** Genetics, Plant genetics, Sequencing, Molecular biology, Transcriptomics

## Abstract

*Populus wulianensis* is an endangered species endemic to Shandong Province, China. Despite the economic and ornamental value of this species, few genomics and genetic studies have been performed. In this study, we performed a relevant analysis of the full-length transcriptome sequencing data of *P*. *wulianensis* and obtained expressed sequence tag (EST)-simple sequence repeat (SSR) markers with polymorphisms that can be used for further genetic research. In total, 8.18 Gb (3,521,665) clean reads with an average GC content of 42.12% were obtained. From the corrected 64,737 high-quality isoforms, 42,323 transcript sequences were obtained after redundancy analysis with CD-HIT. Among these transcript sequences, 41,876 sequences were annotated successfully. A total of 23,539 potential EST-SSRs were identified from 16,057 sequences. Excluding mononucleotides, the most abundant motifs were trinucleotide SSRs (47.80%), followed by di- (46.80%), tetra- (2.98%), hexa- (1.58%) and pentanucleotide SSRs (0.84%). Among the 100 designed EST-SSRs, 18 were polymorphic with high *PIC* values (0.721 and 0.683) and could be used for analyses of the genetic diversity and population structure of *P. wulianensis*. These full-length transcriptome sequencing data will facilitate gene discovery and functional genomics research in *P. wulianensis*, and the novel EST-SSRs developed in our study will promote molecular-assisted breeding, genetic diversity and conservation biology research in this species.

## Introduction

*Populus wulianensis* is an endangered species endemic to Shandong Province, China, with high economic and ornamental value^1,2^, and similar to other species of the genus *Populus*, it is an important source of timber. The effective number of wild survivors of *P. wulianensis* is less than 1000, and thus, this species should be considered among China’s plant species with extremely small populations (PSESP)^3^. There are different views on the taxonomic status of *P*. *wulianensis*. Liang and Li reported *P*. *wulianensis* as an independent species in 1986 and thought that it might have originated from the cross between *Populus adenopoda* and *Populus davidiana*^4^. However, Zhang et al. suggested that *P*. *wulianensis* is an intraspecific variation of *P*. *adenopoda*^5^. At present, taxonomists generally recognize the taxonomic independence of the species^1,2,6,7^. Very few studies have investigated this species, and the previous studies have only focused on its taxonomy, the establishment of regeneration systems and the control of vitrification in test-tube seedlings^5,8^. As a result, few studies have provided molecular data for this endangered species.

In recent years, a large number of molecular markers, such as amplified fragment length polymorphisms (AFLPs), random amplified polymorphic DNA (RAPD), single primer amplification reaction (SRAP), and simple sequence repeats (SSRs), have been developed and applied in the fields of genomic mapping, molecular-assisted breeding, DNA fingerprinting, genetic diversity and population structure analysis, and conservation biology^9–11,12^. Among these molecular markers, SSRs are more reliable and widely used in the above-mentioned research fields due to their abundance in the genome, high polymorphism rate, high information content and codominance^13,14^. SSR markers are divided into two types, namely, expressed sequence tag (EST)-SSRs and genomic SSRs (gSSRs), which originate from RNA transcriptome sequencing and arbitrary genome sequencing, respectively. Compared with than gSSRs, EST-SSRs are functional molecular markers with the advantages of easier and more efficient development, lower cost and more interspecific transferability^15–18^. EST-SSRs developed from transcriptome sequences have also been used in studies of related species^19,20^.

The acquisition of genetic information is the basis for understanding and further studying a species^21–23^. In contrast to whole-genome sequencing, transcriptome sequencing is more cost effective and suitable for genomics research in nonmodel plants, hybrids and some controversial species^24–26^. In contrast to general second-generation sequencing technology, third-generation full-length transcriptome sequencing based on PacBio single-molecule real-time (SMRT) sequencing technology has the advantages of a long read length and high accuracy^27,28^. This technology can directly yield single-molecule full-length mRNA information without the need for assembly and can accurately identify features such as alternative splicing, variable polyadenylation (APA), fusion genes, gene families, and noncoding RNAs^29–31^. Thus, it can meet the requirements of genetic studies and analyses of nonmodel plants. In recent years, full-length transcriptome sequencing studies of some plants, such as *Dendrobium officinale*^32^, *Abrus precatorius*^33^, and *Pogostemon cablin*^34^, have been reported. In addition, the published studies have shown that the obtained transcriptome sequences contain large numbers of EST-SSRs^14,35^. EST-SSRs developed based on transcriptome sequencing data can be used in the genetic analysis of both a sequenced species and its related species and in conservation biology research and molecular-assisted breeding^17,25,36,37^.

The objectives of this study were to (1) obtain full-length transcriptome information for *P. wulianensis* based on PacBio SMRT sequencing technology and perform functional annotation of the transcriptome; and (2) screen a large number of EST-SSRs, design 100 pairs of EST-SSRs for amplification experiments, identify polymorphic primers and characterize their polymorphisms. The full-length transcriptome data obtained in this study will lay a foundation for further genetic analysis of *P. wulianensis* and will be helpful for the discovery and functional annotation of new genes, mapping and molecular-assisted breeding. Furthermore, the polymorphic primers screened and validated in this study will support further research on the conservation biology of *P. wulianensis* and on the genetic relationships among *P. wulianensis* and its related species.

## Results

### Full-length transcriptome sequencing

The transcriptome is an essential tool for understanding life processes. In this study, the full-length transcriptome sequence of *P. wulianensis* was obtained based on SMRT sequencing. In total, 8.18 Gb (3,521,665) of clean reads with an average GC content of 42.12% were obtained (Table 1). The mean clean read length was 2177 (Table 1). We screened a total of 289,128 reads of insert (ROIs) from the original sequence data (Table 1). The mean quality of the ROIs in each library was greater than 0.9 (Table 1). As shown in Table 1, increases in the size of the inserted fragment were associated with gradual decreases in the percentage of full-length sequences to ROI sequences (FLP).

**Table 1:** Full-length transcriptome sequencing data.

cDNA size (kb)	CD	MCDL	ROI	MRLI	MRQI	NFNR	AFNRL	FLP (%)
1–2	1,629,787	1799	128,208	1956	0.93	78,124	1509	61.21
2–3	1,314,986	2364	99,284	2143	0.93	57,066	2106	57.63
3–6	576,892	3708	61,636	3623	0.91	32,875	3488	53.55
All	3,521,665	2177	289,128	2664	0.92	16,8065	7103	57.46

*cDNA size* size of the inserted fragment used to build the library, *CD* clean data, *MCDL* mean length of clean data, *ROI* read of insert, *MRLI* mean read length of insert, *MRQI* mean read quality of insert, *NFNR* number of full-length nonchimeric reads, *AFNRL* average full-length nonchimeric read length, *FLP* full-length percentage.

The Iso-Seq module of the SMRT Analysis software was used to perform a cluster analysis of the above-mentioned full-length sequences. A total of 87,004 consensus isoforms with an average length of 17,642 were obtained (Table 2). Combined with the non-full-length sequences, the quiver program was used to correct the consensus isoforms in each cluster, and 64,737 corrected high-quality isoforms with an accuracy higher than 99% were obtained (Table 2). As shown in Table 2, the highest percentage of polished high-quality isoforms (84.61%) was obtained with a sequence length of 0–1 kb. In contrast, if the sequence size was higher than 6 kb, the percentage of polished high-quality isoforms was only 5.52%. From the corrected 64,737 high-quality isoforms, 42,323 transcript sequences suitable for further analysis were obtained through redundancy analysis with CD-HIT (Supplementary Table S2).

**Table 2:** ICE clustering statistics.

Size (kb)	NCI	ACIRL	NPHI	PPHI (%)
0–1	2176	915	1839	84.51
1–2	44,027	1547	35,739	81.18
2–3	21,372	2331	15,787	73.87
3–6	19,103	3629	11,354	59.44
Above 6	326	9220	18	5.52
All	87,004	17,642	64,737	60.90

*Size* length range of sequence statistics, *NCI* number of consensus isoforms, *ACIRL* average consensus isoform length, *NPHI* number of polished high-quality isoforms, *PPHI* percent of polished high-quality isoforms.

### Functional annotation of transcript sequences

The obtained 42,323 nonredundant transcript sequences were aligned to the following databases using BLAST software (version 2.2.26) (Supplementary Table S2): RefSeq nonredundant proteins (NR), Swiss-Prot Protein Sequence (Swiss-Prot), Gene Ontology (GO), Cluster of Orthologous Groups of proteins (COG), Clusters of orthologous groups for eukaryotic complete genomes (KOG), evolutionary genealogy of genes: Nonsupervised Orthologous Groups (eggNOG), Pfam protein families (Pfam), and Kyoto Encyclopedia of Genes and Genomes (KEGG). A total of 41,876 nonredundant transcript sequences were favorably annotated (Supplementary Table S2). In total, 41,851 transcript sequences were aligned with the NR database (Supplementary Table S2), and among these, 35,070 (83.80%) transcript sequences showed higher than 90% similarity (Supplementary Fig. S1), whereas only 0.45% of the transcript sequences showed less than 50% similarity (Supplementary Fig. S1).

Sequence alignment was used to identify homologous species. As shown in Supplementary Fig. S2, 22,776 (54.42%) transcript sequences were annotated to *Populus trichocarpa*, whereas 14,486 (34.61%) transcript sequences were annotated to *Populus euphratica*. Notably, only 4.03% of the transcript sequences were annotated to *Populus tomentosa*, and 6.03% of the transcript sequences were annotated to plants outside the genus *Populus*.

**Figure 1:**
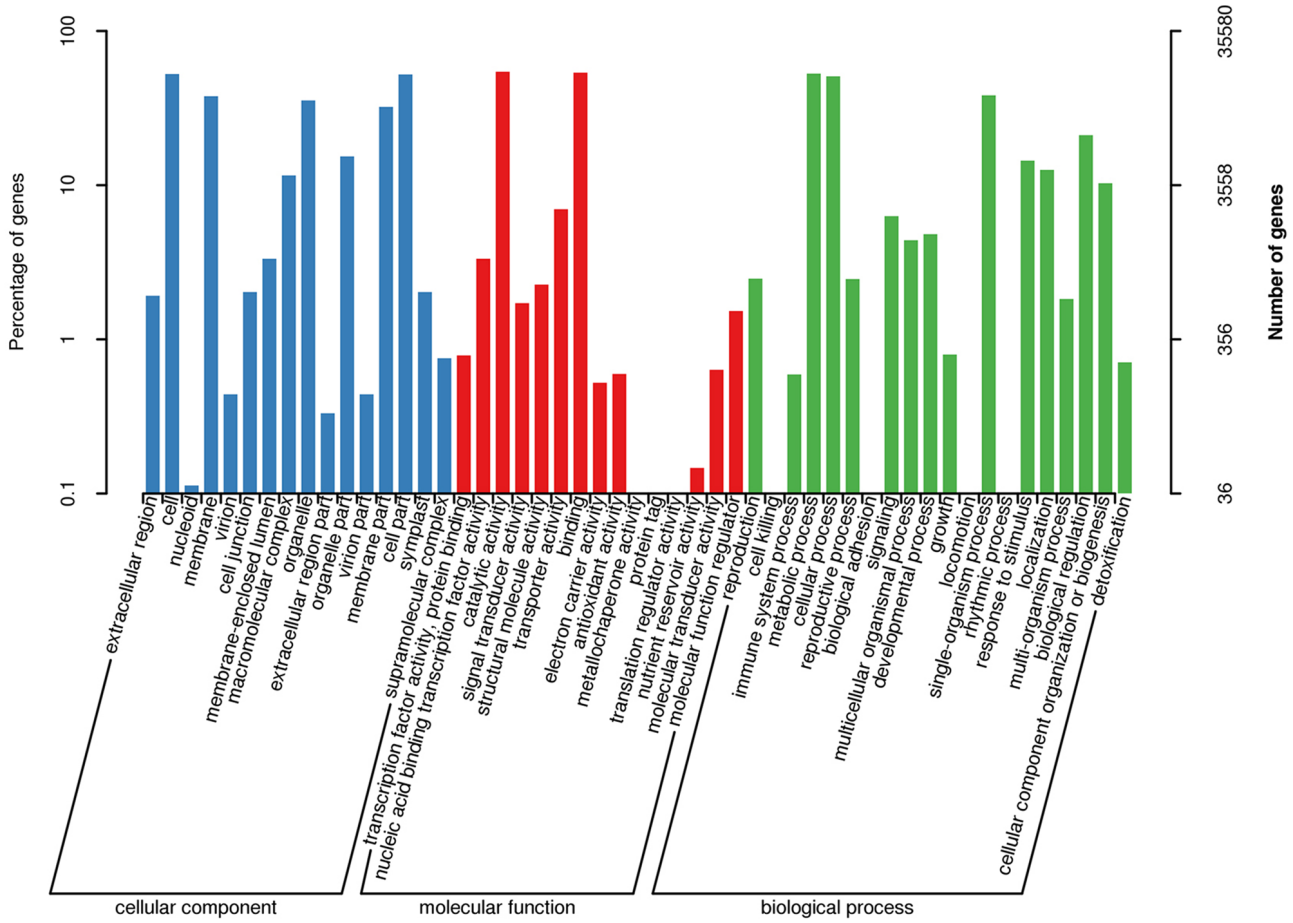
GO annotations of *P. wulianensis* transcript sequences.

The GO database provides a set of dynamically updated standard vocabularies to comprehensively describe the functional attributes of genes and gene products in organisms. In our study, a total of 35,580 transcript sequences were annotated in the GO database and assigned to 51 subcategories within the cellular component (84,429, 41.38%), molecular function (45,154, 21.13%) and biological process (80,133, 37.50%) categories in the GO database (Supplementary Table S2, Fig. 1). In the cellular component category, cell (18,706, 8.75%) constituted the largest group of transcript sequences, followed by cell part (18,613, 8.71%), membrane (13,424, 6.28%), and organelle (12,611, 5.90%). Only 40 (0.02%) transcript sequences were assigned to nucleoids (Supplementary Table S2, Fig. 1). Similarly, in the molecular function ontology, transcript sequences involved in catalytic activity (19,420, 9.09%) formed the largest group, followed by binding (19,135, 8.95%) and transporter activity (2,490, 1.17%) (Supplementary Table S2, Fig. 1). Fewer than 10 transcript sequences were found to be involved in metallochaperone activity (4), protein tag (3), and translation regulator activity (2) (Supplementary Table S2, Fig. 1). In the biological process category, the largest group of transcript sequences was assigned to metabolic processes (18,826, 8.81%), followed by cellular processes (18,132, 8.48%) and single-organism processes (13,626, 6.38%) (Supplementary Table S2, Fig. 1). In contrast, subclasses such as developmental process (1717, 0.80%) and multicellular organismal process (1566, 0.73%) in the biological process category were assigned to less than 1% of the total transcript sequences (Supplementary Table S2, Fig. 1).

**Figure 2:**
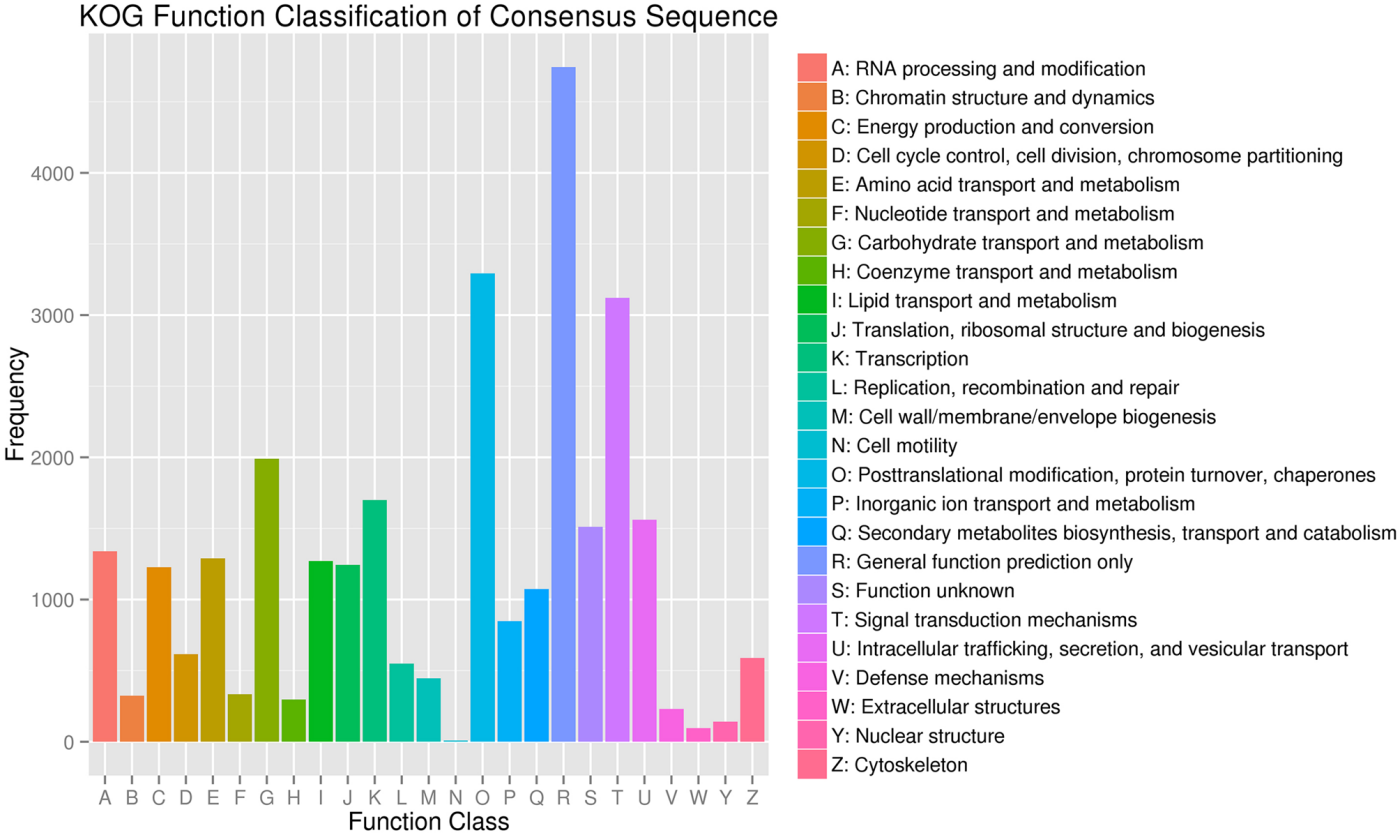
KOG functional classification of *P. wulianensis* transcript sequences.

The KOG, COG and eggNOG databases are all databases for orthologous gene function annotation. The KOG database is based on orthologous relationships among genes and uses evolutionary relationships to divide homologous genes from different species into different ortholog clusters. The COG database is an earlier database that was used to identify orthologous genes and to classify gene products by homology. The eggNOG database is mainly used for functional description annotation and the functional classification of orthologous groups. In our study, a total of 29,851, 20,657, and 41,710 unique transcript sequences were assigned to 25 KOG categories, 26 COG categories, and 25 eggNOG categories, respectively (Supplementary Table S2). Among the 25 KOG categories, the greatest number of transcript sequences was assigned to general function prediction only (4743, 15.89%), followed by posttranslational modification, protein turnover, chaperones (3293, 11.03%) and signal transduction mechanisms (3121, 10.64%) (Supplementary Table S2, Fig. 2). Extracellular structures and cell motility formed the two smallest groups, with 96 (0.32%) and 10 (0.03%) transcript sequences, respectively (Supplementary Table S2, Fig. 2).

Among the 26 COG categories, approximately 30% of the transcript sequences were annotated as general function prediction only (2166, 10.49%), signal transduction mechanisms (2097, 10.15%), and carbohydrate transport and metabolism (2079, 10.06%) (Supplementary Table S2, Supplementary Fig. S3). The results showed that no transcript sequences were annotated as nuclear structure (Supplementary Table S2, Supplementary Fig. S3).

Unlike the KOG and COG categories, no transcript sequence was annotated to general function prediction only when using the eggNOG categories (Supplementary Table S2, Supplementary Fig. S4). The largest number of transcript sequences were annotated to function unknown (18,793, 45.06%), followed by posttranslational modification, protein turnover, chaperones (3280, 7.86%) and transcription (2889, 6.93%) (Supplementary Table S2, Supplementary Fig. S4). With the exception of unknown function, no class contained more than 10% of the transcripts (Supplementary Table S2, Supplementary Fig. S4).

**Figure 3:**
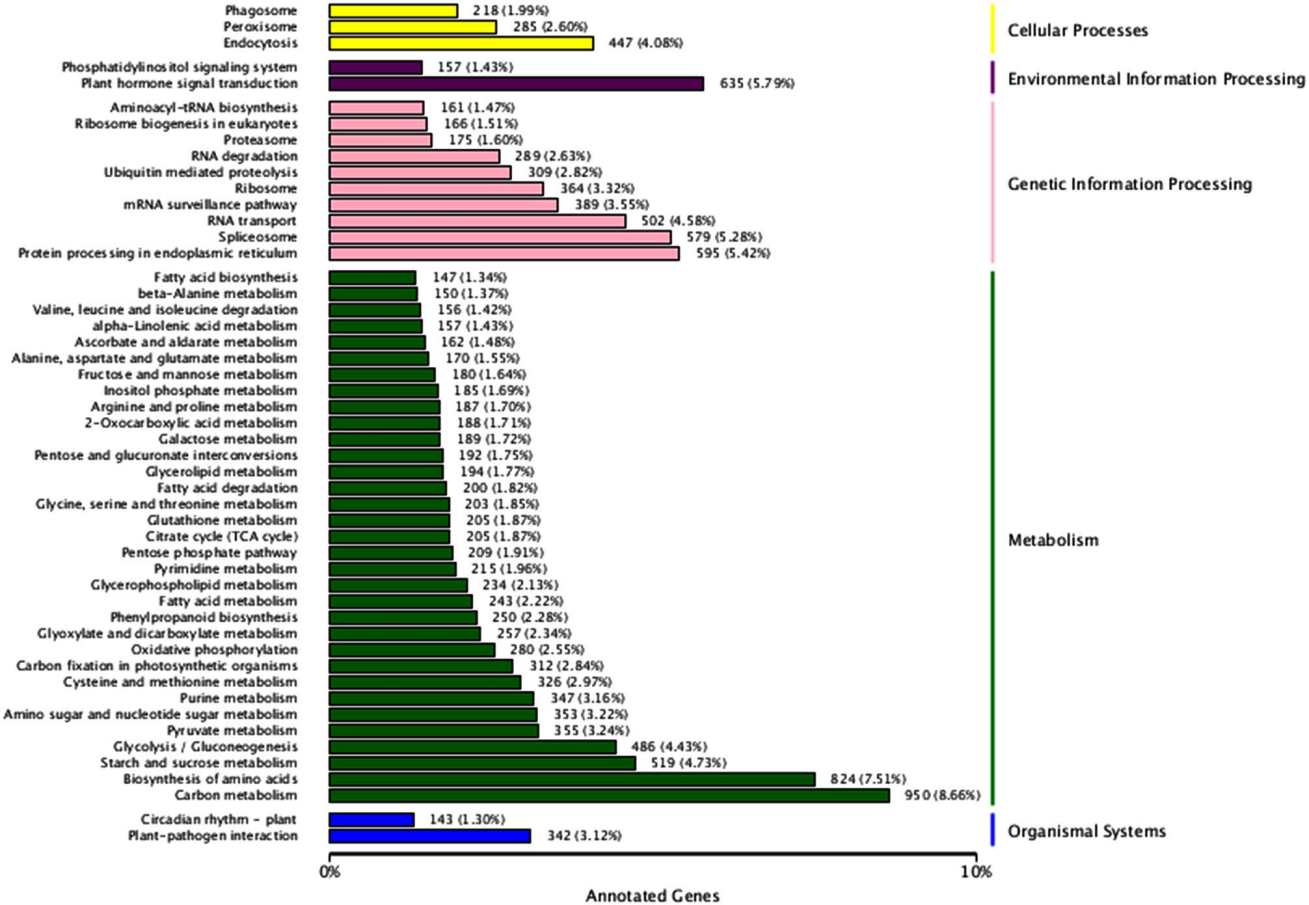
KEGG metabolic categories in the *P. wulianensis* transcriptome.

The pathway annotation analysis of expressed genes helps increase the understanding of gene functions, and KEGG is a powerful tool for in vivo metabolic analysis and metabolic network research. In this study, a total of 19,686 transcript sequences were associated with 128 KEGG pathways in six categories, namely, cellular processes, environmental information processing, genetic information processing, human diseases, metabolism and organismal systems (Supplementary Table S2, Fig. 3). Among the six categories, the category involving the most unigenes was metabolism (13,075, 66.4%) with 96 KEGG pathways, followed by genetic information processing (4230, 21.49%) with 21 KEGG pathways (Supplementary Table S2, Fig. 3). The remaining four categories included only 2381 unigenes, approximately 12% of the total (Supplementary Table S2, Fig. 3).

### Characteristics of SSRs in the transcriptome

Transcripts longer than 500 bp were screened using MIcroSAtellite identification tool (MISA). Among the 42,311 evaluated sequences, we identified 16,057 transcript sequences that contained EST-SSRs. As shown in Table 3, a total of 23,539 SSRs (including 12,520 mononucleotide repeats) were identified from the abovementioned 16,057 SSR-containing sequences. One SSR site was found per 2.64 kb (23,539 SSR loci within 89,101,859 bp). The identification results showed that a total of 5,050 transcript sequences contained more than one EST-SSR locus (Table 3), and a total of 2506 SSRs were present in compound form. In this study, the highest number of identified SSRs (excluding mononucleotide repeats) were trinucleotide repeats (5267, 47.80%), followed by dinucleotide repeats (5157, 46.80%) and tetranucleotide repeats (328, 2.98%) (Table 3). The rarest type of EST-SSR was the pentanucleotide type (93, 0.84%) and not the hexanucleotide type (174, 1.58%) (Table 3). The SSR density results showed that the repeat type with the highest distribution density was trinucleotide (excluding mononucleotide repeats and compound SSRs), with an average of 47.34 SSR loci per Mb, followed by dinucleotide, tetranucleotide, hexanucleotide, and pentanucleotide repeats, with averages of 44.01, 2.93, 1.66, and 0.83 SSR loci per Mb, respectively (Table 3, Supplementary Fig. S5).

**Table 3:** SSR analysis statistics.

Searching item	Numbers	Percentage	SSR density
Total number of sequences examined	42,311	–	–
Total size of examined sequences (bp)	89,101,859	–	–
Total number of identified SSRs	23,539	–	–
Number of SSR-containing sequences	16,057	–	–
Number of sequences containing more than 1 SSR	5050	–	–
Number of SSRs present in compound form	2506	–	–
Mononucleotide	12,520	–	–
Dinucleotide	5157	46.80	44.01
Trinucleotide	5267	47.80	47.34
Tetranucleotide	328	2.98	2.93
Pentanucleotide	93	0.84	0.83
Hexanucleotide	174	1.58	1.66

Among all the nucleotide repeats (excluding mononucleotide repeats), hexanucleotide repeats (74, 56.92%) included the most repeat types, and the main repeat types included AACAGC/CTGTTG (12, 0.11%), AAAAAC/ATTTGT (8, 0.07%), and ACCGCC/CGGTGG (7, 0.06%) (Table 4). The tetranucleotide repeats (23, 17.69%) and pentanucleotide repeats (19, 14.62%) also included multiple repeat types (Table 4). Although the four types of dinucleotide repeats, AG/CT (3788, 34.38%), AT/AT (774, 7.02%), AC/GT (570, 5.17%), and CG/CG (25, 0.23%), only accounted for 3.08% of all repeat types, the proportion of SSRs that were dinucleotide sequences was as high as 46.80% (Tables 3 and 4). Among all SSR repeat types (excluding mononucleotide repeats), the highest number was obtained for AG/CT dinucleotide repeats, accounting for 34.38% of all repeat motifs, followed by AAG/CTT (1138, 10.33%) and AGC/CTG (1078, 9.78%) (Table 4).

**Table 4:** Repeat type and proportion of SSRs.

Searching item	Number of repeat types	Major repeat type	Percentage
Dinucleotide	4	AG/CT (3788, 34.38%), AT/AT (774, 7.02%), AC/GT (570, 5.17%), CG/CG (25, 0.23%)	3.08
Trinucleotide	10	AAG/CTT (1138, 10.33%), AGC/CTG (1078, 9.78%), AGG/CCT (735, 6.67%), ACC/GGT (616, 5.59%), AAT/ATT (528, 4.79%)	7.69
Tetranucleotide	23	AAAG/CTTT (72, 0.65%), AGGG/CCCT (66, 0.60%), AAAT/ATTT (58, 0.53%), AAGG/CCTT (25, 0.23%), ACAT/ATGT (16, 0.15%)	17.69
Pentanucleotide	19	AAAAG/CTTTT (22, 0.20%), AGAGG/CCTCT (17, 0.15%), AAGAG/CTCTT (10, 0.09%), AAAAT/ATTTT (7, 0.06%), AGGGG/CCCCT (6, 0.05%)	14.62
Hexanucleotide	74	AACAGC/CTGTTG (12, 0.11%), AAATAC/ATTTGT (8, 0.07%), ACCGCC/CGGTGG (7, 0.06%), AAAAAT/ATTTTT (6, 0.05%), ACCATC/ATGGTG (6, 0.05%)	56.92

Among all SSR tandem repeats (excluding mononucleotide repeats), the most common tandem repeat number was 6 (3055, 27.72%), followed by 5 (2980, 27.04%) and 7 (1622, 14.72%) (Supplementary Table S4). The number of loci with at least 16 tandem repeats was 197, accounting for 1.79% of all SSR tandem repeats (Supplementary Table S4).

### Verification of novel and polymorphic EST-SSRs

The development of primers constitutes the basis for further research on the genetic structure and diversity of species. Twelve samples, including ten *P. wulianensis* samples, one *P. adenopoda* sample and one *P. davidiana* sample, were subjected to PCR amplification using 100 pairs of newly developed EST-SSRs. Among the 100 EST-SSRs, 12 failed to generate a product, and the other 88 primer pairs successfully resulted in amplification (Supplementary Table S5). Of these 88 primer pairs, 31 exhibited poor universal applicability, eight produced multiple bands, and 12 were monomorphic (Supplementary Table S5). Of the remaining 37 primer pairs capable of generating polymorphic amplification products, 19 primer pairs generated unstable and unclear amplification, and the remaining 18 produced stable and clear amplification products (Supplementary Table S5). Details of these 18 primer pairs can be found in Supplementary Table S6.

**Figure 4:**
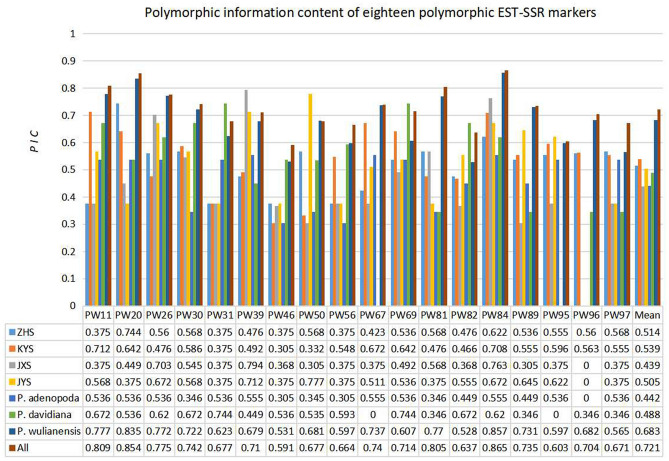
*PIC* values of 18 polymorphic EST-SSR markers.

To further verify the polymorphism of these 18 primer pairs, we performed amplification experiments using 30 samples from six populations, including 24 *P*. *wulianensis* samples, three *P*. *adenopoda* samples and three *P*. *davidiana* samples, and a more accurate high-performance capillary electrophoresis method. The amplification results obtained for all the samples showed that a total of 150 alleles were observed, and the number of alleles (*N*_*A*_) ranged from 5 to 15 per locus, with an average allele number of 8.333 (Supplementary Table S7). Their polymorphism information content (*PIC*) values ranged from 0.591 to 0.865, with an average of 0.721 (Fig. 4). The average values of observed heterozygosity (*H*_*O*_) and expected heterozygosity (*H*_*E*_) were 0.772 and 0.775, respectively, and these values ranged from 0.261 to 0.966 and from 0.637 to 0.877, respectively (Supplementary Table S7). The amplification results from only the 24 samples of *P. wulianensis* detected a total of 117 alleles, and the number of alleles ranged from 3 to 13, with an average *N*_*A*_ of 6.50 (Supplementary Table S7). The *PIC* values ranged from 0.528 to 0.857, with an average of 0.683 (Fig. 4). The average *H*_*O*_ and *H*_*E*_ values were 0.772 and 0.775, respectively (Supplementary Table S7). The amplification results obtained from the samples of the six populations are shown in Supplementary Table S7.

## Discussion

Transcriptome research is one of the essential tools for understanding the life processes of species^38^. Based on next-generation sequencing (NGS), researchers have performed a large number of gene expression and quantitative studies of *Populus* species^39–41^. Zhang et al. characterized cDNA libraries of mature xylem from tension wood (72.17%), opposite wood (70.13%), and normal wood (73.58%) of *P*. *tomentosa*, and their results showed that the length of most of the transcripts (more than 70%) was approximately 500–1000 bp^42^. In our study, a total of 8.18 Gb (3,521,665) of clean reads was obtained. The length of high-quality transcripts (approximately 97.16%) obtained in this study was almost greater than 1000 bp (Table 2), and this transcript length was clearly significantly higher than that obtained previously with second-generation sequencing^42^, which suggests that third-generation full-length transcriptome sequencing based on PacBio SMRT sequencing technology can compensate for the limitation of the short reading length observed with NGS and promote further in-depth study of *P*. *wulianensis* and its related species without reference genomes.

In our study, a total of 41,876 nonredundant transcript sequences were successfully annotated with the functional databases (Supplementary Table S2). These annotated sequences lay a foundation for further studies of genetic differentiation in *P*. *wulianensis*. It has been previously reported that *P*. *tomentosa* best matches *P*. *trichocarpa* in the NR database^41^, and the species-based annotation performed in the present study also showed that *P*. *wulianensis* was most closely related to *P*. *trichocarpa*. However, in contrast to *P*. *trichocarpa* (sect. *Tacamahaca*), *P*. *wulianensis* and *P*. *tomentosa* both belong to sect. *Populus*, which indicates that the homology between *P*. *wulianensis* and *P*. *tomentosa* is higher than that between *P*. *wulianensis* and *P*. *trichocarpa* or *P*. *tomentosa* and *P*. *trichocarpa*. Notably, only 4.03% of the transcript sequences were annotated to *P*. *tomentosa* in our study (Supplementary Fig. S2). This contradiction is most likely due to the scarcity of reports on the transcriptome of species that belong to sect. *Populus*. Furthermore, the results of the present study showed that the number of annotations of *P*. *wulianensis* transcripts obtained with the GO and KEGG databases was significantly higher than that obtained for *P*. *tomentosa* (16,774 and 11,670, respectively)^41^. This finding is mainly attributed to the fact that the PacBio SMRT sequencing technology was used to obtain third-generation full-length transcriptomes in this study. It has been reported that the isoform sequencing (Iso-Seq) of transcriptomes by PacBio is advantageous for genome annotation^43^. Overall, the annotation information provided in our study is more sufficient than that obtained based on NGS and thus increases our understanding of the active biological and metabolic processes in *P*. *wulianensis*. Obviously, the transcript data obtained in our study solved the problem of the lack of a reference genome for this species and will provide rich annotation information for the more convenient identification of specific expression and the more precise quantification of gene and transcript expression in *P*. *wulianensis* and its related species.

SSRs are well known and widely used in studies of the genetic diversity and population structure of species^44–46^. Compared with gSSRs, EST-SSRs are functional molecular markers with the advantages of easier and more efficient development, lower cost and more interspecific transferability^15–17^. Furthermore, EST-SSRs can be used directly to obtain gene expression data due to their close link to functional genes^14^. Previous studies have shown that a large number of potential EST-SSRs are found in transcriptome sequencing data^47,48^. In this study, a total of 23,539 SSRs (including 12,520 mononucleotide repeats) were identified from the abovementioned 16,057 SSR-containing sequences (Table 3). The distribution density, predominant repeat motif, and types of SSRs show differences among different plants^14,48^. In this study, one SSR site was found per 2.64 kb (23,539 SSR loci within 89,101,859 bp), and this SSR density is higher than the density of 1/3.88 kb found through EST-SSRs developed using 20,023 EST sequences from *Populus deltoides* and *Populus euramericana*^49^. Among the SSR repeat types identified in this study, the number of trinucleotide repeat types was the largest (excluding single nucleotide repeats), in contrast to the results of some previous studies in other species, such as *Styrax japonicus* and *Rhododendron latoucheae*^14,17^. However, consistent with the results of some previous studies, such as studies on *Neolitsea sericea*, *P. deltoides* and *P. euramericana*, the AG/CT dinucleotide repeat motif was found at the highest frequency, possibly due to the methylation of cytosine, whereas the CG/CG was detected at the lowest frequency^49,50^. Another possible explanation for the high frequency of the AG/TC repeat in the EST sequence data is that the AG/TC motif can appear in mRNA in the form of GAG, AGA, UCU and CUR codons, which are translated to the amino acids Arg, Glu, Ala and Leu, respectively; Ala and Leu appear in proteins with high frequencies of 8% and 10%, respectively^49^. The distribution frequencies of different types of other nucleotide repeats obtained in previous studies show differences^14,25,37^, and there are various reasons for these differences. Specifically, different species, different searching and development tools, different principles of primer development and design, and the size of the transcript database all might affect the observed SSR distribution frequencies^20,37,47^.

Studies on the genetic diversity of other *Populus* species based on molecular markers have been performed. Specifically, researchers have analyzed the genetic diversity of *P. euphratica* (0.713–0.878), *Populus nigra* (0.51–0.60) and *Populus simonii* (0.589–0.731) using SSR markers, and their results revealed moderate or high *H*_*E*_^51–53^. However, the results from studies on *P. euphratica* (0.059–0.212) and *Populus ilicifolia* (0.091–0.135) using RAPD and AFLP molecular markers, respectively, showed a low level of genetic diversity^54,55^. These reports indicate that SSR exhibits higher polymorphism than the molecular markers RAPD and AFLP, and this difference is mainly attributed to the large variation in the number of SSR repeats. Consistent with previous results on the genetic diversity of *Populus* species, such as *P. euphratica* (0.713–0.878) and *Populus simonii* (0.589–0.731)^51,53^, the 18 EST-SSRs developed in our study for genetic research in *P*. *wulianensis* (0.582–0.852) also exhibited high polymorphism. Interestingly, the *H*_*E*_ values (0.637–0.877) of the three *Populus* species based on the EST-SSR analysis performed in this study were all higher than the *H*_*E*_ (0.475–0.488) of *Populus szechuanica* var. *tibetica*^56^. This comparison revealed that the 18 EST-SSRs had higher polymorphism than those used for *P. szechuanica* var. *tibetica*. In addition, the mean *PIC* value (0.721) of the 18 EST-SSRs used in our study was also higher than that (0.562) of 20 nuclear SSR loci studied in European black poplar^52^. Therefore, the 18 EST-SSRs developed in this study clearly constitute an efficient tool that can be widely used for studying the genetic diversity of *P*. *wulianensis* and its relationship with related species.

## Conclusions

In this study, 8.18 Gb of clean reads were obtained, and the length of high-quality transcripts (approximately 97.16%) obtained in this study was almost greater than 1000 bp. In addition, 41,876 sequences were annotated successfully, and 11,019 EST-SSRs were identified (excluding mononucleotide repeats). Furthermore, 18 EST-SSRs with high polymorphism were verified as reliable molecular marker tools for genetic diversity research in *P*. *wulianensis*. Obviously, these large amount of transcription data will facilitate most genetic analyses of *P*. *wulianensis*, such as the discovery and functional verification of new genes, mapping, and molecular-assisted breeding. The above-mentioned markers will also help reveal the genetic relationship of *P*. *wulianensis* and its related species in terms of functional molecular markers.

## Methods

### Plant materials, DNA and RNA isolation

The plant materials used in this study were obtained from the wild. All the samples were collected with the approval and permission of the local authorities. Based on the protection of wild plant resources, particularly endangered plant resources, we were only collected a small number of plant specimens. During the sample collection and experiment, we strictly abided by China’s laws and regulations regarding the protection of endangered wild plant resources and complied with the Convention on the Trade in Endangered Species of Wild Fauna and Flora. Prof. Dekui Zang formally identified all the samples, and information on the samples and specimens used in this study can be found in Supplementary Table S1.

For transcriptomic analysis, samples from five tissue types (root tips, stems, leaves, buds and male flowers) were collected from vigorously growing and healthy *P. wulianensis* male specimens growing in Kunyu Mountain National Nature Reserve (Supplementary Table S1). Similarly, samples from six tissue types (root tips, stems, leaves, buds, female flowers and fruits) were collected from vigorously growing and healthy *P. wulianensis* female specimens (Supplementary Table S1). All the samples were frozen in liquid nitrogen immediately after collection and stored at − 80 °C until the experiment. The extraction of total DNA and the isolation of total RNA were performed according to Wiland-Szymańskas^57^ and Ghawana^58^, respectively. To ensure the accuracy of the data, the purity, concentration, and nucleic acid absorption peaks of the isolated RNA were detected using a Nanodrop spectrophotometer, and the RNA integrity was accurately tested with an Agilent 2100 instrument. We used electrophoresis to assess the contamination of the RNA samples with genomic DNA.

For the polymorphism analysis of the developed EST-SSRs, the young leaves of ten *P. wulianensis* individuals at Kunyu Mountain, Zhaohu Mountain, Jiuxian Mountain and Juyu Mountain were collected and stored in silica gel (Supplementary Table S1). Similarly, samples of *Populus adenopoda* and *Populus davidiana* from Tianmu Mountain and Culai Mountain, respectively, were collected and stored using the above method (Supplementary Table S1). The modified cetyltrimethylammonium bromide (CTAB) method was used for total genomic DNA extraction^59^.

### cDNA library construction and online sequencing

A cDNA library was constructed using qualified samples. The SMARTer™ PCR cDNA Synthesis Kit was used for the synthesis of full-length cDNA from mRNA. The full-length cDNA fragments were screened using BluePippin and then amplified again by PCR. Subsequently, end repair of the full-length cDNA was performed, and the dumbbell-shaped SMRT adapter was connected. After exonuclease digestion, BluePippin was used for secondary screening to obtain a cDNA library. Qubit 2.0 and Agilent 2100 were used to accurately quantify and detect the library size, respectively. After the libraries passed quality control, full-length transcriptome sequencing was performed using PacBio RSII according to the target offline data volume.

### Analysis of transcriptome sequencing and annotation

The ROI sequences were extracted from the original sequences according to the following criteria: full passes ≥ 0 and sequence accuracy > 0.75. The ROI sequences were divided into full-length and non-full-length sequences based on the presence of the 3′ primer, 5′ primer, and PolyA (optional). The ROI sequences from the same transcript were clustered using the iterative isoform-clustering (ICE) algorithm. ROIs with similar sequences were clustered, and each cluster yielded a consensus sequence. High-quality sequences (accuracy > 99%) were obtained by polishing the consensus sequences using the non-full-length sequences and used for subsequent analysis. CD-HIT was used to remove redundant sequences from the high-quality transcripts to obtain nonredundant sequences (identity > 0.99)^60^. To obtain annotation information for the transcripts, the obtained nonredundant transcript sequences were aligned to the NR, Swiss-Prot, GO, COG, KOG, EggNOG, Pfam, and KEGG databases using BLAST software (version 2.2.26)^61–68^. R version 3.6.1 was used for data analysis^69^.

### EST-SSR detection and primer design

Potential EST-SSRs included in transcript sequences longer than 500 bp were searched and analyzed using the MIcroSAtellite identification tool (MISA). In our study, the SSR loci were identify based on the following criteria: repeat numbers of mono-, di-, tri-, tetra-, penta-, hexa- repeat motifs greater than or equal to 10, 6, 5, 5, 5, and 5, respectively. The maximum number of bases for two SSRs in an interrupted composite microsatellite was 100. EST-SSR primers were designed using Primer 3.0 software. The primer design was performed based on the following principles: (1) the primer length was 18–27 bp, usually 20 bp; (2) the annealing temperature was 57–63 °C, the optimal temperature was 60 °C, and the difference between the Tm values of the upstream and downstream primers did not exceed 5 °C; (3) the GC content was 20–80%, and the optimal GC content was 50%; and (4) the PCR amplification products were expected to have a length of 100–280 bp. The EST-SSR primers were synthesized by Shanghai Biological Engineering (Shanghai) Company.

### Amplification and validation of EST-SSRs

To screen out primers with polymorphisms, the abovementioned 100 primer pairs were synthesized for amplification. PCR was performed in a 20-μL reaction volume, which included 1 μL of template DNA (40 ng/µL), 1 μL of the forward primer (10 μmol/L), 1 μL of the reverse primer (10 μmol/L), 10 μL of 2× EasyTaq PCR SuperMix, and 7 μL of ddH_2_O. PCR amplification was performed using the following temperature program: predenaturation at 94 °C for 5 min; nine cycles of denaturation at 94 °C for 30 s, annealing at 59 °C for 30 s, and extension at 72 °C for 30 s; 21 cycles of denaturation at 94 °C for 30 s, annealing at 55 °C for 30 s, and extension at 72 °C for 30 s; extension at 72 °C for 3 min; and preservation at 4 °C^49^. During the selection of polymorphic primers, the PCR amplification products were run on a vertical plate electrophoresis apparatus using a 6% nondenaturing polyacrylamide gel. In the primer polymorphism verification experiments, high-performance capillary electrophoresis, which has higher accuracy than a nondenaturing polyacrylamide gel, was performed.

## Supplementary information

Supplementary Information 1.

Supplementary Information 2.

Supplementary Information 3.
