# Supplementary material for: Full-length transcriptome sequencing analysis and development of EST-SSR markers for the endangered species Populus wulianensis
Source: Sci Rep. 2020 Oct 1;10:16249. doi: 10.1038/s41598-020-73289-5 (PMC7530656; doi:10.1038/s41598-020-73289-5)
Supplement: Supplementary file 1 — Supplementary Information 1. [file 41598_2020_73289_MOESM1_ESM.pdf]

Supplementary Information

(Supplementary Figure S1 to Figure S8)

(Supplementary Table S1, Table S5, Table S6, Table S7)

Full-length transcriptome sequencing analysis and development of EST-SSR markers for the endangered species *Populus wulianensis*

Qichao Wu1+ Fengqi Zang2+ Xiaoman Xie3 Yan Ma1* Yongqi Zheng2 Dekui Zang1

1 College of Forestry, Key Laboratory of State Forestry Administration for Silviculture of the Lower Yellow River, Shandong Agricultural University, Tai' an 271018, China

2 Institute of Forestry, Chinese Academy of Forestry, Beijing 100091, P. R. China

3 Center for Forest Genetic Resources of Shandong Province, Jinan 250014, China

+ Co-first author, these authors contributed equally to this work

* the corresponding author: myiris@sina.com

(Supplementary Figure S1 to Figure S8)

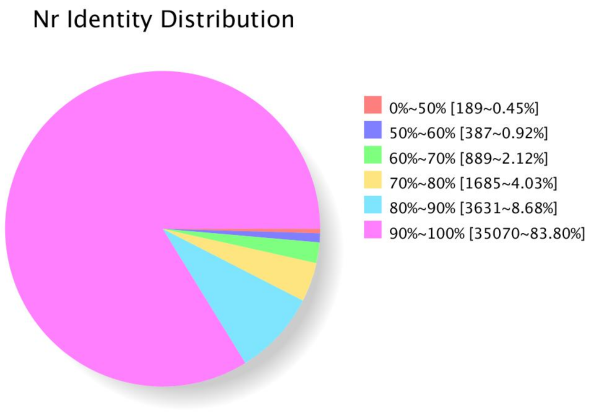

Supplementary Figure S1. Nr identity distribution.

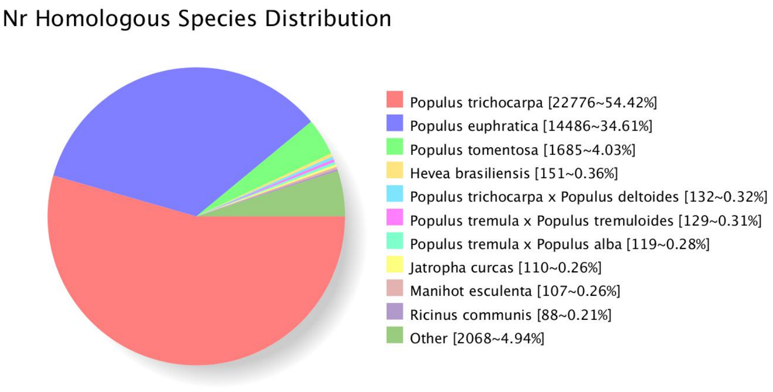

Supplementary Figure S2. Nr homologous species distribution.

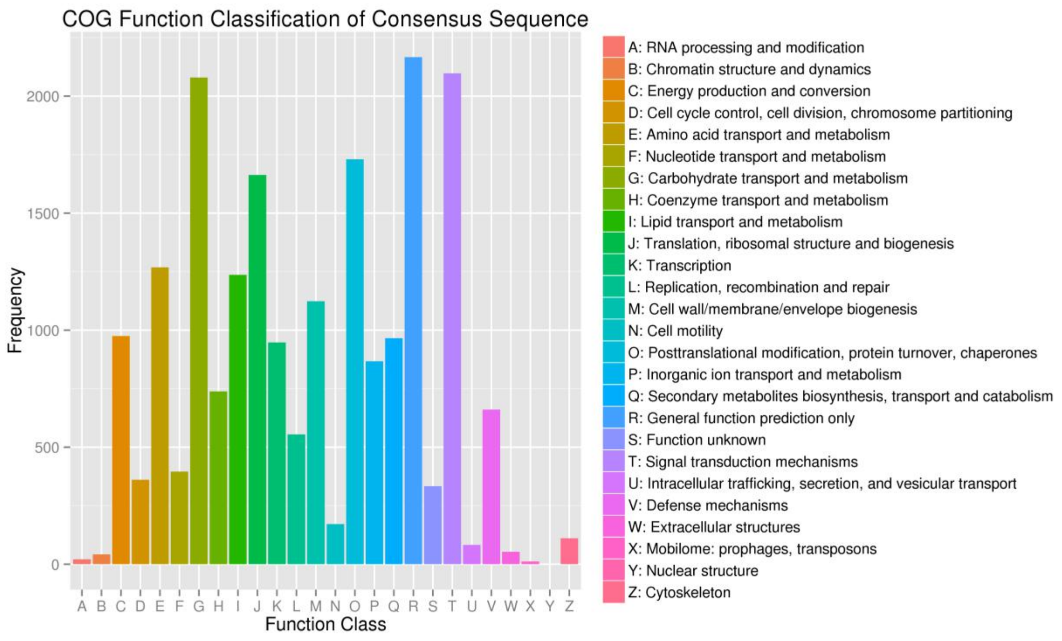

Supplementary Figure S3. COG functional classification of *P. wulianensis* transcript sequences.

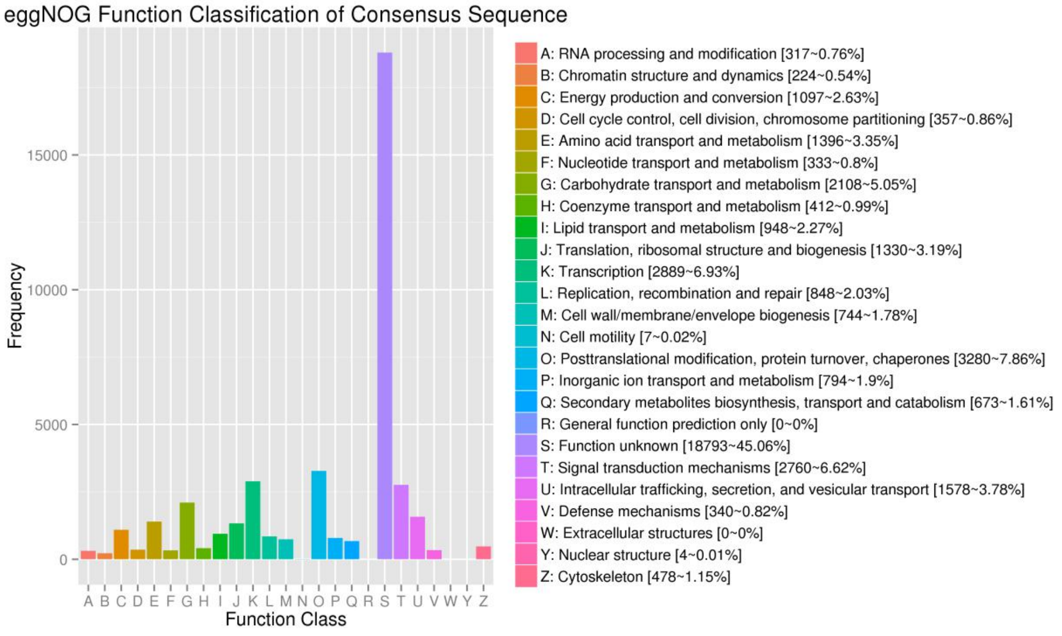

Supplementary Figure S4. EggNOG functional classification of *P. wulianensis* transcript sequences.

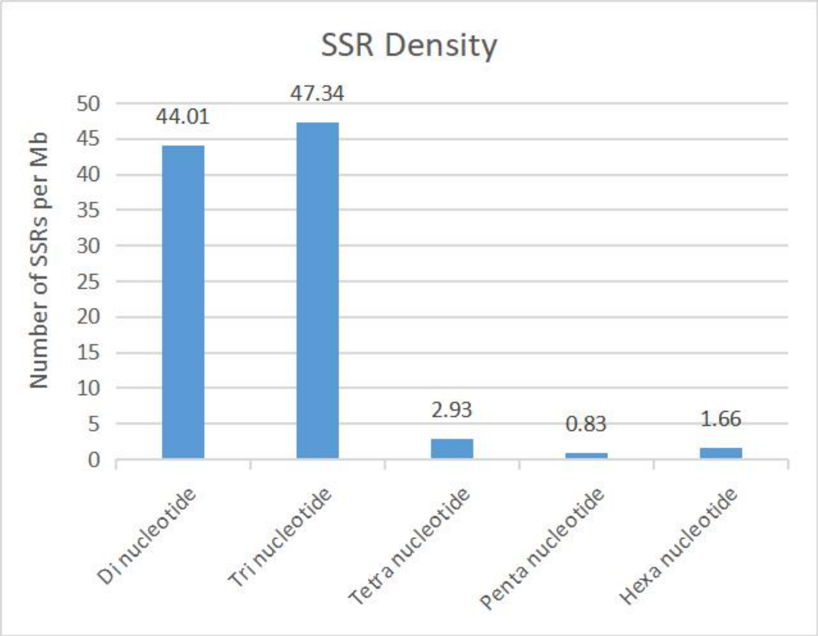

Supplementary Figure S5. SSR density of *P. wulianensis.*

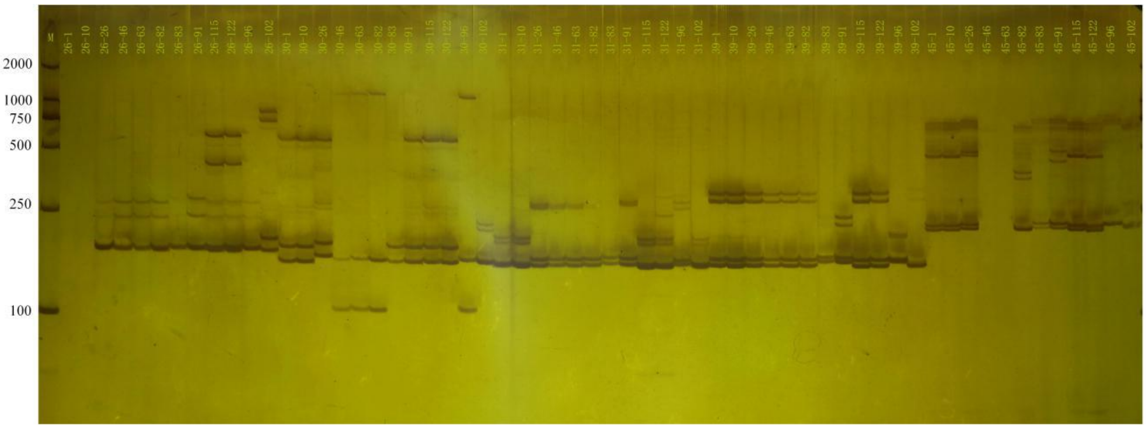

Supplementary Figure S6. Gel photographs of genotyping 1. Note: M- Marker; Primers- 26, 30, 31, 39, 45; Samples- *P. wulianensis* (1, 10, 26, 46, 63, 82, 83, 91, 115, 122), *P. adenopoda* (96), *P. davidiana* (102).

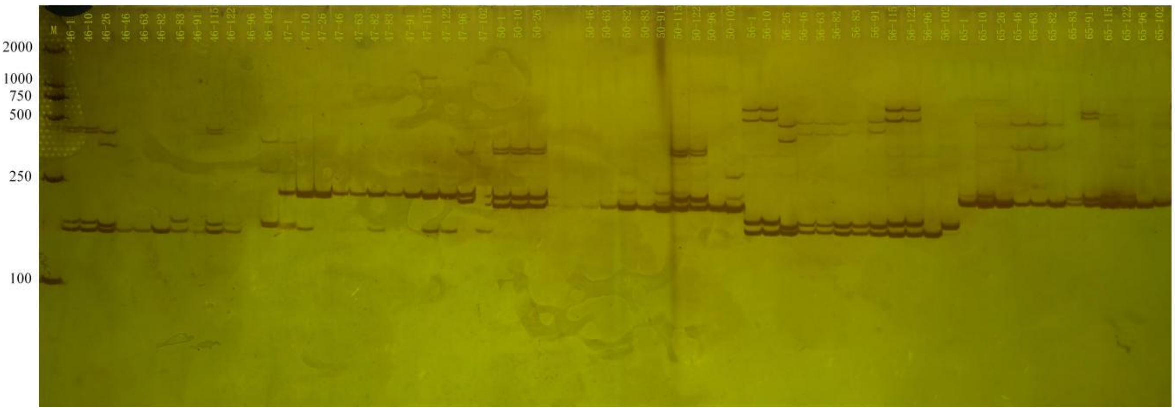

Supplementary Figure S7. Gel photographs of genotyping 2. Note: M- Marker; Primers- 46, 47, 50, 56, 65; Samples- *P. wulianensis* (1, 10, 26, 46, 63, 82, 83, 91, 115, 122), *P. adenopoda* (96), *P. davidiana* (102).

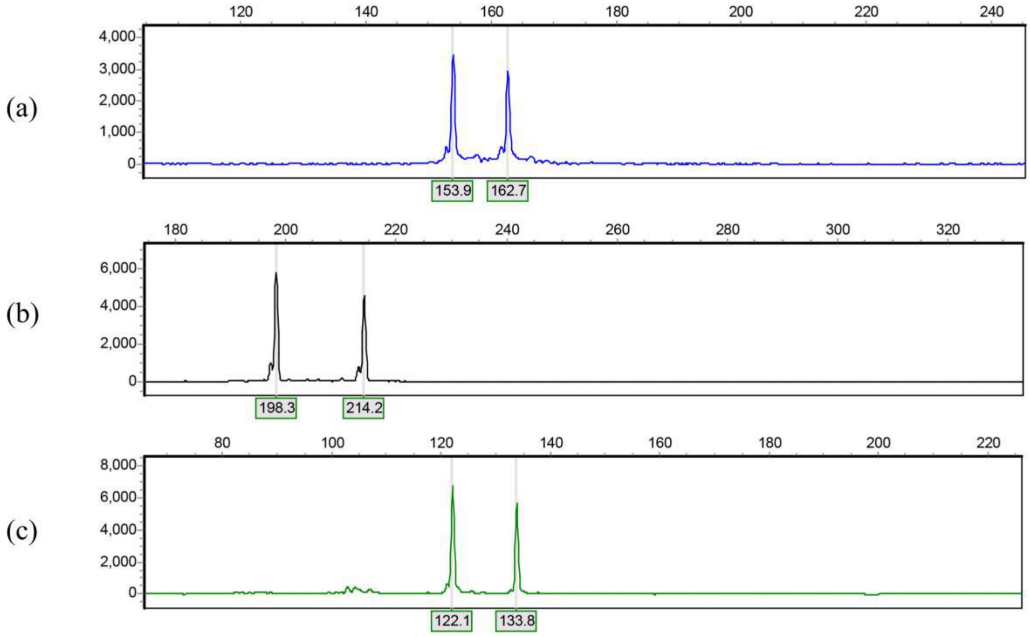

Supplementary Figure S8. Segregations of alleles generated by the primers PW39, PW50 and PW97 of *P. wulianensis*. Note: (a)- Primer PW39; (b)- Primer PW50; (c)- Primer PW97.

(Supplementary Table S1, Table S5, Table S6, Table S7)

Table S1 Information of the samples used in the present study.

Item	Samples	Location	Latitude (°N)	Longitude (°E)	Altitude (m)	Appraiser	Voucher specimen	Storage location
Samples for transcriptomic analysis	*Populus wulianensis* (male specimen)	Mt. Kunyu, Yantai City, Shandong Prov.	37.27446	121.75936	186	Dekui Zang	ZangDK-SDKY-2018-001	SDAU
	Populus wulianensis (female specimen)	Mt. Kunyu, Yantai City, Shandong Prov.	37.27446	121.75936	186	Dekui Zang	ZangDK-SDKY-2018-002	SDAU
Samples for EST-SSRs polymorphism analysis	*Populus wulianensis*	Mt. Zhaohu, Yantai City, Shandong Prov.	36.85928	121.19879	156	Dekui Zang		
	Populus wulianensis	Mt. Zhaohu, Yantai City, Shandong Prov.	36.85920	121.19875	155	Dekui Zang		
	Populus wulianensis	Mt. Zhaohu, Yantai City, Shandong Prov.	36.85952	121.19878	156	Dekui Zang		
	Populus wulianensis	Mt. Zhaohu, Yantai City, Shandong Prov.	36.86063	121.20077	154	Dekui Zang	ZangDK-SDZH-2018-001	SDAU
	Populus wulianensis	Mt. Zhaohu, Yantai City, Shandong Prov.	36.86066	121.20087	155	Dekui Zang		
	Populus wulianensis	Mt. Zhaohu, Yantai City, Shandong Prov.	36.86082	121.20116	156	Dekui Zang		
	Populus wulianensis	Mt. Kunyu, Yantai City, Shandong Prov.	37.27414	121.76046	189	Dekui Zang		
	Populus wulianensis	Mt. Kunyu, Yantai City, Shandong Prov.	37.27405	121.76022	193	Dekui Zang		
	Populus wulianensis	Mt. Kunyu, Yantai City, Shandong Prov.	37.27401	121.75983	199	Dekui Zang		
	Populus wulianensis	Mt. Kunyu, Yantai City, Shandong Prov.	37.27446	121.75800	227	Dekui Zang	ZangDK-SDKY-2017-003	SDAU
	Populus wulianensis	Mt. Kunyu, Yantai City, Shandong Prov.	37.27529	121.75452	306	Dekui Zang		
	Populus wulianensis	Mt. Kunyu, Yantai City, Shandong Prov.	37.27333	121.75771	238	Dekui Zang		
	Populus wulianensis	Mt.Jiuxian, Rizhao City, Shandong Prov.	35.68243	119.35881	372	Dekui Zang		
	Populus wulianensis	Mt.Jiuxian, Rizhao City, Shandong Prov.	35.68243	119.35883	373	Dekui Zang		
	Populus wulianensis	Mt.Jiuxian, Rizhao City, Shandong Prov.	35.68248	119.35872	371	Dekui Zang		

Populus wulianensis	Mt.Jiuxian, Rizhao City, Shandong Prov.	35.68212	119.35917	366	Dekui Zang	ZangDK-SDJX-2017-001	SDAU
Populus wulianensis	Mt.Jiuxian, Rizhao City, Shandong Prov.	35.68218	119.35919	365	Dekui Zang		
Populus wulianensis	Mt.Jiuxian, Rizhao City, Shandong Prov.	35.68217	119.35917	366	Dekui Zang		
Populus wulianensis	Mt.Juyu, Weihai City, Shandong Prov.	36.87389	121.37694	124	Dekui Zang		
Populus wulianensis	Mt.Juyu, Weihai City, Shandong Prov.	36.87389	121.37722	125	Dekui Zang		
Populus wulianensis	Mt.Juyu, Weihai City, Shandong Prov.	36.87389	121.37694	125	Dekui Zang		
Populus wulianensis	Mt.Juyu, Weihai City, Shandong Prov.	36.87361	121.37722	124	Dekui Zang	ZangDK-SDJY-2018-001	SDAU
Populus wulianensis	Mt.Juyu, Weihai City, Shandong Prov.	36.87111	121.37750	123	Dekui Zang		
Populus wulianensis	Mt.Juyu, Weihai City, Shandong Prov.	36.87390	121.37722	125	Dekui Zang		
Populus adenopoda	Mt. Tianmu, Hangzhou City, Zhejiang Prov.	30.32824	119.45341	452	Dekui Zang		
Populus adenopoda	Mt. Tianmu, Hangzhou City, Zhejiang Prov.	30.32925	119.45065	469	Dekui Zang	ZangDK-HZTM-2017-00	SDAU
Populus adenopoda	Mt. Tianmu, Hangzhou City, Zhejiang Prov.	30.32969	119.45081	467	Dekui Zang	1	
Populus davidiana	Mt. Culai, Tai'an City, Shandong	36.05372	117.29620	787	Dekui Zang		
Populus davidiana	Mt. Culai, Tai'an City, Shandong	36.05355	117.29261	792	Dekui Zang	ZangDK-SDCL-2018-001	SDAU
Populus davidiana	Mt. Culai, Tai'an City, Shandong	36.05351	117.29269	792	Dekui Zang		

Mt- mountain; Prov- province; SDAU- Shandong Agricultural University.

Table S5 Amplification results with the 100 EST-SSR markers.

NO.	Amplification results	Number of primers	Percentage(%)
1	No amplification	12	12%
2	Poor universal applicability	31	31%
3	Multiple bands	8	8%
4	Monomorphism	12	12%
5	Unstable and Unclear amplification	19	19%
6	Stable and clear amplification	18	18%

Table S6 Eighteen polymorphic EST-SSR markers.

Primer	Type	SSRs	Forward Primer (5'-3 ')	Tm (°C)	Reverse Primer (5'-3 ')	Tm (°C)	Gene ID
PW11	Di-nucleotide	(GA)13	CAACACCCCCAACTTCATCT	55.0	CGCACAATGAGCTAATCTCG	53.8	F01_c2795/f1p0/1068
PW20		(TG)15	TCTCTCTCTTCCACTCCCCA	57.1	TAGCCACGAAAGCCTAAAGC	55.2	F01_c10080/f3p1/2131
PW26	Tri-nucleotide	(AGA)9	CCCCCAGTATCCCGATCTAT	55.6	AACTGACATGAACTTCCCCG	54.9	F01_c4598/f2p1/1790
PW30		(ATC)12	TCGTAGGTCTCCCTGTTGCT	58.1	GCCGAGCTTGAGCTTGTTAT	55.5	F01_c13558/f1p5/3843
PW31		(ATG)6	TAATCAGGAGCAGCAGCAGA	56.0	GAGCAGAGACCAAAAGGCAC	56.7	F01_c16660/f1p0/2075
PW39		(GCC)7	CCTCCACCACTCTCATCTCC	57.4	AGAGCCACGAGTCAAGGAAA	56.1	F01_c13084/f1p3/1371
PW46	Tetra-nucleotide	(CCTC)7	ACCGCATCTAAAACGGACAC	55.4	GAGGAAATTAACAACCGCCA	52.8	F01_c38049/f2p2/1750
PW50		(TCCC)8	ACGGCGCTCTGAACTGATA	56.3	TGATCAGACCCACCAAACAA	53.7	F01_c610/f1p1/954
PW56		(AAGA)5	TCCATGGAAAACGAATCCTC	52.0	GCCCATGTTGGTGTTTCTTC	54.5	F01_c2199/f3p0/1869
PW67	Penta-nucleotide	(GTTTT)6	AATGATTCCAGCCGATGAAG	52.4	CCCTTGTCATGGCTACGATT	54.8	F01_c14871/f1p0/2387
PW69		(CTCCT)6	CATCCCATCTCCTCCACTTC	55.0	AAAAACCCACTTTGGTGCTG	53.8	F01_c22425/f15p13/2163
PW81	Hexa-nucleotide	(CCGCCA)5	AAAAGACCAAAACAACCCCA	52.0	GCACGGGCAAGAGAAGATAG	56.0	F01_c1837/f3p0/1348
PW82		(TCCCTC)5	GCTTCCAGAGTCTGCAGAGAA	56.9	TCCTGAAAAGACCAGCGAGT	56.1	F01_c7659/f1p0/1523
PW84		(CGCCAC)8	ATCACCACCACCACCTCTTC	57.2	TTTGTGGGTTTTTGAGGGAG	52.7	F01_c13779/f2p1/1781
PW89		(GACAAG)5	CTCAACCGAAGCTGAACCTC	56.1	TGAAAACGGCCATATGTTGA	51.6	F01_c28730/f1p2/1316
PW95		(GGCGAA)5	TGGATGGGAGGTTTAGGTTG	54.3	AGCCCAAGTTCTCTGCAAAA	54.5	F01_c9174/f2p1/2674
PW96		(TGGAAC)5	TCACTTGGTTTGGGTGTTGA	54.1	GTTTTTCAAAAGCTCCGACG	52.2	F01_c16585/f1p0/2332
PW97		(GGCATG)6	CCTACGATTGGTTCTGGCAT	54.8	TGCATTCCTCCATAACCTCC	54.3	F01_c1002/f6p5/3467

Table S7. Characterization of 18 polymorphic EST-SSR markers for *P. wulianensis.*

Pops \ Primer		PW11	PW20	PW26	PW30	PW31	PW39	PW46	PW50	PW56	PW67	PW69	PW81	PW82	PW84	PW89	PW95	PW96	PW97	Mean
ZHS	N_A	2	5	4	4	2	3	2	4	2	3	3	4	3	4	3	3	4	4	3.278
	H_O	1.000	1.000	1.000	1	1.000	1.000	1.000	1.000	1.000	0.667	1.000	1.000	1.000	1.000	1.000	1.000	0.667	1.000	0.963
	H_E	0.500	0.778	0.625	0.639	0.500	0.569	0.500	0.639	0.500	0.486	0.611	0.639	0.569	0.681	0.611	0.625	0.625	0.639	0.596
	PIC	0.375	0.744	0.560	0.568	0.375	0.476	0.375	0.568	0.375	0.423	0.536	0.568	0.476	0.622	0.536	0.555	0.560	0.568	0.514
KYS	N_A	5	5	4	3	2	3	2	2	3	4	5	3	3	5	3	4	3	3	3.444
	H_O	1.000	0.500	0.500	0.600	1.000	1.000	0.500	0.600	1.000	1.000	0.500	1.000	0.800	0.667	1.000	1.000	0.400	1.000	0.782
	H_E	0.750	0.681	0.514	0.660	0.500	0.580	0.375	0.420	0.620	0.722	0.681	0.569	0.540	0.750	0.625	0.660	0.640	0.625	0.606
	PIC	0.712	0.642	0.476	0.586	0.375	0.492	0.305	0.332	0.548	0.672	0.642	0.476	0.466	0.708	0.555	0.596	0.563	0.555	0.539
JXS	N_A	2	3	4	3	2	6	2	2	2	2	3	4	2	6	2	2	1	2	2.778
	H_O	0.000	0.000	1.000	0.833	1.000	1.000	0.833	0.500	1.000	1.000	1.000	1.000	0.833	0.833	0.000	1.000	0.000	1.000	0.713
	H_E	0.500	0.500	0.750	0.625	0.500	0.820	0.486	0.375	0.500	0.500	0.580	0.639	0.486	0.792	0.375	0.500	0.000	0.500	0.524
	PIC	0.375	0.449	0.703	0.545	0.375	0.794	0.368	0.305	0.375	0.375	0.492	0.568	0.368	0.763	0.305	0.375	0.000	0.375	0.439
JYS	N_A	4	2	4	4	2	6	2	6	2	3	3	2	3	4	4	4	1	2	3.222
	H_O	1.000	1.000	1.000	1.000	1.000	1.000	1.000	1.000	1.000	1.000	1.000	1.000	1.000	1.000	1.000	1.000	0.000	1.000	0.944
	H_E	0.639	0.500	0.722	0.639	0.500	0.750	0.500	0.806	0.500	0.594	0.611	0.500	0.625	0.722	0.700	0.681	0.000	0.500	0.583
	PIC	0.568	0.375	0.672	0.568	0.375	0.712	0.375	0.777	0.375	0.511	0.536	0.375	0.555	0.672	0.645	0.622	0.000	0.375	0.505
P. wulianensis	N_A	8	10	7	7	4	9	4	6	4	7	6	9	3	13	5	4	6	5	6.500
	H_O	0.800	0.625	0.864	0.870	1.000	1.000	0.818	0.810	1.000	0.900	0.870	1.000	0.913	0.875	0.810	1.000	0.316	1.000	0.860

	H_E	0.804	0.852	0.801	0.758	0.681	0.721	0.582	0.724	0.651	0.765	0.656	0.796	0.606	0.870	0.769	0.660	0.727	0.636	0.726
	PIC	0.777	0.835	0.772	0.722	0.623	0.679	0.531	0.681	0.597	0.737	0.607	0.770	0.528	0.857	0.731	0.597	0.682	0.565	0.683
P. adenopoda	N_A	3	3	3	2	3	3	2	2	2	3	3	2	3	3	3	3	1	3	2.611
	H_O	0.333	0.667	0.333	0.000	0.667	1.000	0.500	0.000	0.500	0.500	0.667	0.667	0.333	0.500	0.333	0.333	0.000	1.000	0.463
	H_E	0.611	0.611	0.611	0.444	0.611	0.625	0.375	0.444	0.375	0.625	0.611	0.444	0.500	0.625	0.500	0.611	0.000	0.611	0.513
	PIC	0.536	0.536	0.536	0.346	0.536	0.555	0.305	0.345	0.305	0.555	0.536	0.346	0.449	0.555	0.449	0.536	0.000	0.536	0.442
P. davidiana	N_A	4	3	4	4	5	3	3	3	3	1	5	2	4	4	2	1	2	2	3.056
	H_O	0.333	0.333	0.667	0.667	1.000	0.333	0.667	0.333	0.667	0.000	1.000	0.000	0.333	0.667	0.667	0.000	0.000	0.000	0.426
	H_E	0.722	0.611	0.667	0.722	0.778	0.500	0.611	0.611	0.667	0.000	0.778	0.444	0.722	0.667	0.444	0.000	0.444	0.444	0.546
	PIC	0.672	0.536	0.620	0.672	0.744	0.449	0.536	0.535	0.593	0.000	0.744	0.346	0.672	0.620	0.346	0.000	0.346	0.346	0.488
All	N_A	11	12	9	9	8	11	5	7	6	7	8	9	6	15	8	5	7	7	8.333
	H_O	0.692	0.600	0.786	0.759	0.966	0.926	0.778	0.667	0.920	0.760	0.862	0.867	0.793	0.828	0.741	0.792	0.261	0.900	0.772
	H_E	0.829	0.867	0.802	0.773	0.722	0.745	0.637	0.721	0.704	0.772	0.746	0.827	0.690	0.877	0.765	0.662	0.742	0.717	0.755
	PIC	0.809	0.854	0.775	0.742	0.677	0.710	0.591	0.677	0.664	0.740	0.714	0.805	0.637	0.865	0.735	0.603	0.704	0.671	0.721

ZHS, the test samples of *P. wulianensis* collected from Zhaohu Mountain; KYS, the test samples of *P. wulianensis* collected from Kunyu Mountain; JXS, the test samples of *P. wulianensis* collected from Jiuxian Mountain; JYS, the test samples of *P. wulianensis* collected from Juyu Mountain; *P. wulianensis,* the test samples of *P. wulianensis* collected from Zhaohu Mountain, Kunyu Mountain, Jiuxian Mountain, Juyu Mountain; *P. adenopoda*, the test samples of *P. adenopoda* collected from Tianmu Mountain; *P. davidiana*, the test samples of *P. adenopoda* collected from Culai Mountain; All, All of the above test samples including *P. wulianensis*, *P. adenopoda*, and *P. davidiana*; N_A, total number of alleles at that locus; H_O, observed Heterozygosity; H_E, expected Heterozygosity; PIC, polymorphic information content.
